# Genotype-phenotype correlation in Pompe disease, a step forward

**DOI:** 10.1186/s13023-014-0102-z

**Published:** 2014-08-08

**Authors:** Paola De Filippi, Kolsoum Saeidi, Sabrina Ravaglia, Andrea Dardis, Corrado Angelini, Tiziana Mongini, Lucia Morandi, Maurizio Moggio, Antonio Di Muzio, Massimiliano Filosto, Bruno Bembi, Fabio Giannini, Giovanni Marrosu, Miriam Rigoldi, Paola Tonin, Serenella Servidei, Gabriele Siciliano, Annalisa Carlucci, Claudia Scotti, Mario Comelli, Antonio Toscano, Cesare Danesino, The Italian GSDII Group

**Affiliations:** Department of Molecular Medicine, University of Pavia, Pavia, Italy; IRCCS Neurological Institute C. Mondino, Pavia, Italy; Regional Centre for Rare Diseases, University Hospital Santa Maria della Misericordia, Udine, Italy; IRCCS San Camillo, Lido Venice, Italy; Centre for Neuromuscular Diseases “P. Peirolo”, Department of Neurosciences, University of Torino, Torino, Italy; Immunology and Muscular Pathology Unit, National Neurological Institute “Carlo Besta”, Milan, Italy; Department of Neurology, Fondazione IRCCS Ca’ Granda Ospedale Policlinico, Centro Dino Ferrari, University of Milan, Milan, Italy; Centre for Neuromuscular Disease, CeSI, University ‘G. d’Annunzio’, Chieti, Italy; Clinical Neurology, Section for Neuromuscular Diseases and Neuropathies, University Hospital Spedali Civili, Brescia, Italy; Department of Medical, Surgical and Neurological Sciences, University of Siena, Siena, Italy; Neuromuscular Unit, Department of Cardiovascular Science and Neurology, University of Cagliari, Sardinia, Italy; Rare Metabolic Diseases Unit, Fondazione MBBM San Gerardo Hospital, Monza, Italy; Department of Neurological Sciences and Vision, Section of Neurology, University of Verona, Verona, Italy; Department of Neurology, Università Cattolica, Rome, Italy; Neurological Institute, University of Pisa, Pisa, Italy; Department of Pulmonary rehabilitation, Fondazione IRCCS S. Maugeri, Pavia, Italy; Department of Brain and Behavioural Sciences, University of Pavia, Pavia, Italy; Department of Neurosciences, Psychiatry and Anaesthesiology, University of Messina, Messina, Italy

**Keywords:** Glycogen storage disease type II, Genetic polymorphisms, Modifier genes, GAA, ACE, ACTN3

## Abstract

**Background:**

Pompe’s disease is a progressive myopathy caused by mutations in the lysosomal enzyme acid alphaglucosidase gene (*GAA*). A wide clinical variability occurs also in patients sharing the same *GAA* mutations, even within the same family.

**Methods:**

For a large series of GSDII patients we collected some clinical data as age of onset of the disease, presence or absence of muscular pain, Walton score, 6-Minute Walking Test, Vital Capacity, and Creatine Kinase. DNA was extracted and tested for *GAA* mutations and some genetic polymorphisms able to influence muscle properties (*ACE, ACTN3, AGT and PPARα* genes).

We compared the polymorphisms analyzed in groups of patients with Pompe disease clustered for their homogeneous genotype.

**Results:**

We have been able to identify four subgroups of patients completely homogeneous for their genotype, and two groups homogeneous as far as the second mutation is defined “very severe” or “potentially less severe”. When disease free life was studied we observed a high significant difference between groups. The DD genotype in the *ACE* gene and the XX genotype in the *ACTN3* gene were significantly associated to an earlier age of onset of the disease. The *ACE* DD genotype was also associated to the presence of muscle pain.

**Conclusions:**

We demonstrate that ACE and ACTN3 polymorphisms are genetic factors able to modulate the clinical phenotype of patients affected with Pompe disease.

**Electronic supplementary material:**

The online version of this article (doi:10.1186/s13023-014-0102-z) contains supplementary material, which is available to authorized users.

## Background

Glycogen Storage Disease type II (GSDII) or Pompe disease (OMIM 232300) is a lysosomal storage disorder caused by mutations in the acid alpha-glucosidase (*GAA*) gene whose frequency is about 1 in 40000–50000 in European and US Populations and apparently much lower in Australia or Portugal [1]. A comprehensive review of clinical and genetic data was published by van der Ploeg and Reuser [2]. More than 300 different mutations have been described [3], without strict genotype-phenotype correlations [4]. Even within families large variations in disease severity can be observed; a recent report by Wens et al. [5] demonstrates as presenting symptoms were different in sibling in 8/22 (36%) families. The clinical heterogeneity of GSDII is more frequently observed among late-onset patients (i.e. childhood, juvenile and adult onset) than among early-onset cases [6], it is observed even within families [6,7] and is reported also in animal models [8].

The same mutation can be observed in both infantile and late onset patients even if with different incidence; for instance, in two different groups of Italian patients, the c.525delT was observed in 13,8% in cases with the infantile form [9] but also in 3,8% of cases of late onset disease [10]. Conversely the same authors reported an incidence of 3,4% for the c.2237G > A mutation in infants as compared to an incidence of 10,3% in adults.

A possible exception is the c.-32-13T > G mutation which never occurs in patients with the classic infantile form.

This mutation was first reported by Huie et al. [11] and then demonstrated to be common in late onset patients by many groups as Montalvo et al. [12], Gort et al. [13], Kroos et al. [14] and Joshi et al. [15]; it allows the production of normally spliced GAA mRNA in a very small amount, and the production of tree splicing variants [16,17]. The presence of even a limited amount of normal mRNA has been interpreted as the cause of delayed onset of the disease in patients carrying this mutation [15].

However genotypes including the c.-32-13T > G plus a second mutation still show a very variable clinical presentation.

The extensive variability discussed prompted several authors to suggest that other (genetic) factors may consistently influence the clinical outcome of the disease [4,5].

The polymorphism of Angiotensin converting enzyme (*ACE*) had been previously reported as genotype modulator in McArdle disease, also a glycogen storage disease, by Martinuzzi et al. 2003; they reported that the number of D alelles was significantly correlated with severity of phenotype.

Thus we decided to evaluate if also in Pompe’s disease the different *ACE* polymorphism could modulate the final phenotype of the disease. As *ACE* polymorphism is relevant also in muscle fiber type composition and athletic performances, we included in our study two more genes whose polymorphisms may also act on the same parameters, as alpha actinin 3 (ACTN3) and peroxisome proliferative activated receptor alpha(PPARα); Angiotensinogen (AGT) was finally added as its protein is the precursor of the substrate for ACE.

We then studied a large sample of late-onset GSDII patients, all sharing the c.-32-13T > G mutation and divided them in subgroups based on the second mutation.

Our aim was to elucidate whether groups of cases homogeneous for the presence of the same couple of *GAA* mutations but differing in some genetic polymorphisms may show distinct clinical features as for: age of onset, muscle pain, muscle and respiratory dysfunction, serum creatine kinase (CK).

## Methods

### Patients and clinical methods

Thanks to the collaboration with the GSDII - Italian Group, we recruited a large series of GSDII patients (n = 126, 58 males; age range 10–83) as described in Angelini et al. [18].

The patients were diagnosed in many different centres and no evidence that index cases sharing the same mutations were related was recorded.

Informed consents were obtained according to the rules of the centres caring for the patients, who also provided clinical data. All the work was carried on in compliance with the Helsinki Declaration.

We collected the following data: age of onset of the disease (defined as the age the patient first notices difficulties in running/climbing stairs), thus disease free survival is the time from birth to the age of symptom onset; presence or absence of muscular pain at any time after the diagnosis; Walton score [19]; 6-Minute Walking Test (6MWT) [20]; Vital Capacity, as expressed as percentage of decrease compared to controls (VC%); creatine kinase (CK).

Walton score, 6MWT, and VC were the latest available data for patients not on ERT and the latest estimation prior to starting ERT for the others. As for CK, we reported the levels measured at the time of diagnosis; due to inhomogeneous reference values across laboratories, we calculated a ratio by dividing each patient’s CK by the upper limit of normal range of that site’s local laboratory. Physicians who collected clinical data were blind to genetic data.

### Molecular analysis

Among all patients in whom both mutations were identified in our three labs (C.D, C.A. and M.M.), we selected 85 cases carrying the c.-32-13T > G on one allele.

The definition of “very severe (VS)” or “potentially less severe (PLS)” is as in http://www.pompecenter.nl; the severity rating of mutations is according to [21] based on size, amount and enzymatic activity of mutated protein expressed in transfected COS-7 cells.

The genetic polymorphisms were analysed by P. De F. and K.S.

DNA was extracted by routine methods using the “GENE ELUTE” kit by Sigma, amplification of all *GAA* exons was performed according to published methods [22].

### Polymorphisms analyzed

1. The angiotensin converting enzyme (*ACE)* polymorphism is characterized by the presence (insertion (I)) or absence (deletion (D)) of a 287-base-pair alu repeat within intron 16 [23].
2. The R577X polymorphism in alpha-actin-3 (*ACTN3)* is a C to T transversion at position 1,747 in exon 16 [24].
3. The Peroxisome proliferator-activated receptor alpha (*PPARα)* polymorphism is located in intron 7 (456 bp upstream of exon 8) and is a G to C substitution [25].
4. The polymorphism of the angiotensinogen (*AGT*) gene is a T to C transition at base 704 resulting in Met235Thr [26].

The methods for polymorphism analysis were reported by Lindpainter et al. [23] (*ACE*), North et al. [24] (*ACTN3*), Flavell et al. [25] (*PPARα*); Gomez-Gallego et al. [26] (*AGT*).

### Statistical methods

The independence of categorical variables was tested by the X^2^ test. The comparisons of scores among groups were made by the Kruskal-Wallis test.

The disease free survival of the different Allele 2 groups was compared by the logrank test when the proportional hazard assumption was granted. The survival curves shown are estimated by the Kaplan-Maier (K-M), or the K-M like method when the application of a Cox model was possible.

The disease free life of the polymorphisms as well as of their interaction was analyzed by lognormal parametric accelerated life survival models, which optimally fitted the data.

### Structural modelling

A model of GAA was built with the M4T server v. 3.0 (Bioinformatics. (2007) 23, 2558–65; Nucleic Acids Res. (2007) 35, W363-68; J. Struct. Funct. Genomics. (2009) 10,95-9). The submitted sequence included the full-length GAA sequence (UNIPROT number P10253, residues 1–952). Templates used were chain As of human maltase-glucoamylase (2QLY and 3L4T). Dope score (Shen MY, Sali A. “Statistical potential for assessment and prediction of protein structures” Protein Sci. 2006 Nov;15(11):2507–24) was −108411 and PROSA 2003 ZSCORE was −13.08 (Sippl,M.J. “Recognition of Errors in Three-Dimensional Structures of Protein, Proteins, 17: 355–362 (1993)). Images were generated with Pymol (The PyMOL Molecular Graphics System, Version 1.5.0.4 Schrödinger, LLC).

## Results

We collected a large series of patients with non classic forms of Pompe disease, all carrying the c.-32-13T > G mutation, a mutation which is common in these forms of the disease. As to the second mutation, we have been able to identify four subgroups of patients completely homogeneous for their genotype, in details their genotypes were: c.-32-13T > G/c.525delT; c.-32-13T > G/c.2237G > A; c.-32-13T > G/Delexon18; c.-32-13T > G/c.1927G > A. Finally two groups were defined “homogeneous” as far as the second mutation is defined “very severe” or “potentially less severe” according to the database http://www.pompecenter.nl (Table 1). In addition 21 mutations other than those reported in Table 1, were observed in more than one patient, but the small size of this subgroups prevented further correlation with clinical features; these mutations are listed in Additional file 1: Table S1.

**Table 1:** **Frequency of the different mutations observed in our sample**

**Mutations Allele 1 Allele 2**		**N° of pts.**	**%**
c.-32-13T > G (N = 85)	c.525delT	21	24,7
	c.2237G > A	13	15,3
	Delexon18*	7	5,8
	c.1927G > A	5	5,8
	Very severe (VS)	13	17,6
	Potentially less severe (PLS)	26	30,5

*Delexon18 is also referred in the literature as c.2481 + 102_2646 + 31del.

### Correlation between GAA genotypes and clinical parameters

#### Disease-free life

We collected the information on the age of onset of the disease (and thus on the disease free life) in all 85 patients.

We compared the disease-free survival between five genotypes all of them showing the c.-32-13T > G as first allele and carrying a different mutation on the second allele (c.-32-13T > G/c.525delT; c.-32-13T > G/c.2237G > A; c.-32-13T > G/Delexon18; c.-32-13T > G/c.1927G > A; c.-32-13T > G/VS and c.-32-13T > G/PLS as described in Table 1), and a significant difference was observed: p = 0.031. We also compared the genotypes in which the c.-32-13T > G was associated to a mutation classified as “very severe” (VS) with those in which IVS1 was associated to a mutation classified as “potentially less severe” (PLS) and no significant difference was found between the two groups (P = 0.231) (data not shown).

The c.525delT group was excluded from the comparison because it does not satisfy the proportional hazard assumption (see statistical methods).

**Figure 1:**
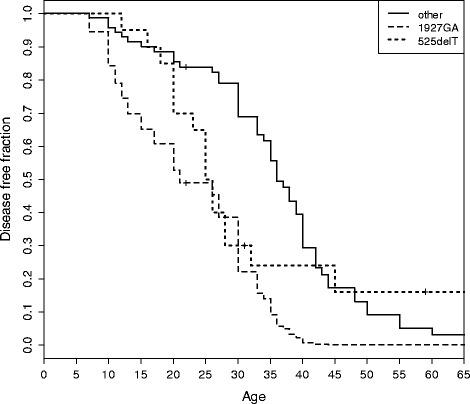
**Comparison of disease-free life among patients with genotypes c.-32-13T > G/c.525delT (n = 21), c.-32-13T > G/c.1927G > A (n = 5), c.-32-13T > G/other (c.2237G > A, Delexon18, VS, PLS) (n = 59).** The curve for c.-32-13T > G/c.525delT is a Kaplan-Mayer estimate, the other two are pseudo K-M estimates derived from a Cox model.

Figure 1 shows the estimated disease-free survival for the c.525delT group, the c.1927G > A group and the whole of the other groups; the c.-32-13T > G/c.1927G > A group shows a shorter disease-free survival compared to the other groups (p = 0.000163).

**Figure 2:**
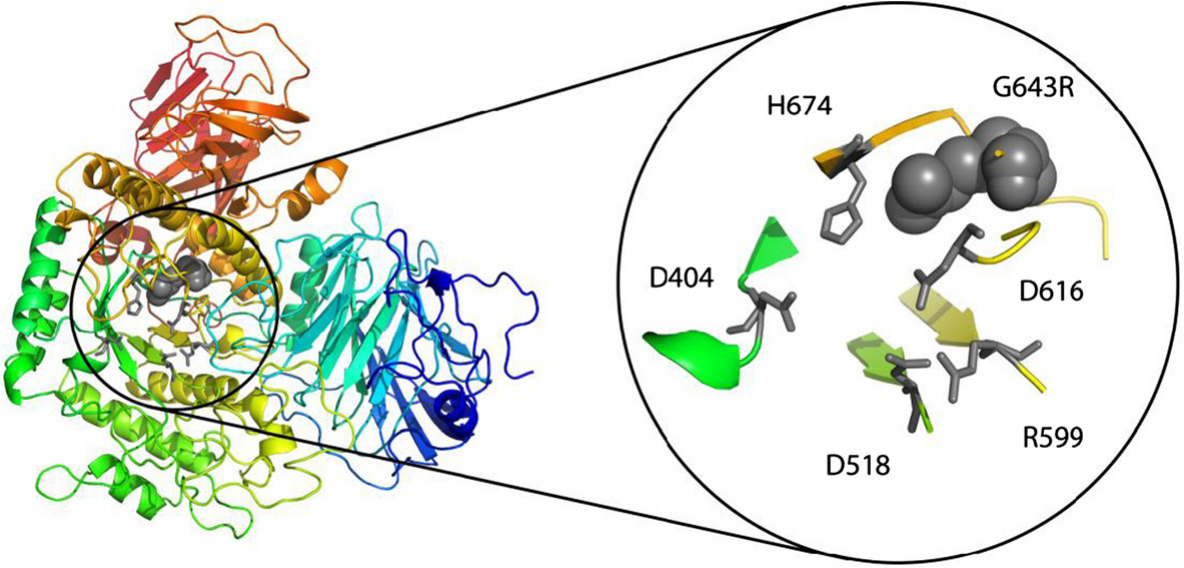
**Left: structural model of GAA.** Right: magnification of the catalytic site. Grey sticks: catalytic residues. Grey spheres: G643R mutation (c.1927G > A).

The c.1927G > A mutation is a missense mutation, resulting in the p.Gly643Arg and as the genotype c.-32-13T > G/c.1927G > A behaves differently, we analyzed where the substituded aminoacid, Arginin, is placed in the structure of the molecule (Figure 2).

Other clinical features studied were similar through different *GAA* genotypes, as reported in Table 2.

**Table 2:** **Summary of clinical and biochemical data assessed in the different Allele 2 groups, and statistical analysis (p)**

**Allele 2**	**Presence of pain, % [68]**	**Walton score**	**6 MWT**	**VC %**	**CK**
		**Median, (IQR), [77]**	**Median, (IQR), [43]**	**Median, (IQR), [43]**	**Median, (IQR), [40]**
c.525delT	38.9 [7/18]	2.25 (2 – 3.1) [20]	313 (196–386 ) [9]	61.0 (49.0 - 71.0) [9]	3.3 (2.3 - 4.0) [8]
c.2237G > A	33.3 [ 3/9]	2.25 (1.2 -4.5) [10]	144 (65–244) [4]	87.5 (59.7 - 89.0) [6]	1.35 (0.7 - 3.1) [6]
Delexon18	20.0 [1/5]	1.0 (3–0) [5 ]	378 (194–432) [5]	67.0 (56.0 -78.0) [5]	0.8 (0.8 - 1.5) [5]
c.1927 G > A	20.0 [1/5]	3.0 (2–0) [5 ]	307 (199–333) [3]	57.5 (47.7- 65.5) [4]	3.0 (1.9 - 6.3) [3]
VS	33.3 [4/12]	3.0 (4–0) [13]	292 (208–375) [2]	43.5 (32.7 - 54.2) [2]	2.8 (1.7 - 3.8) [2]
PLS	52.6 [10/19]	3.0 (4–0) [24]	368 (256–394) [12]	68.1 (63.0 - 79.3) [17]	1.8 (1.5 - 3.0) [16]
p	0,655	0,7021	0.493	0.461	0.170

In brackets [ ] the number of cases for which the data are available. IQR: interquartile range.

### Correlation between ACE, ACTN3, AGT and PPARα polymorphisms and clinical parameters

As the polymorphisms in the genes studied (ACE, ACTN3, AGT and PPARα) are relevant for some muscular physiological and structural functions, we analyzed whether a correlation with some clinical parameters could be observed.

The observed distribution of the polymorphisms in our sample did not differ from that reported in controls (ACE [23], ACTN3 [27], AGT [26], and PPARα [28]) see Table 3.

**Table 3:** **Distribution of the different polymorphisms in our sample as compared to data reported in controls**

	***ACE***	***ACTN3***	***AGT***	***PPARα***
	**DD ID II**	**RR RX XX**	**TT TC CC**	**GG GC CC**
Patients	23 46 15	25 44 15	23 33 17	43 32 5
Controls	29 37 14	35 78 41	41 59 19	322 161 5
Number of cases:	85	84	74	80
Patients controls	80	154	119	500
Xsquare test, p	P = 0,448	p = 0.238	p = 0.474	p = 0.136

Hypothesis tested: cases and controls have the same distribution.

No difference was found for age, gender and disease duration for any of the different subgroups identified by the polymorphisms studied for any gene (data not shown); we report only details about ACE and ACTN3 as they are the only genes whose polymorphisms showed some significant differences (see below). Analysis for age, gender and disease duration in patients with DD/ID/II genotype for ACE gave respectively the following p values (Kruskal-Wallis): 0.31; 0.41 and 0.76. Analysis for age, gender and disease duration in patients with XX/RX/RR genotype for ACTN3 gave respectively the following p values (Kruskal-Wallis): 0.59; 0.44 and 0.78.

**Figure 3:**
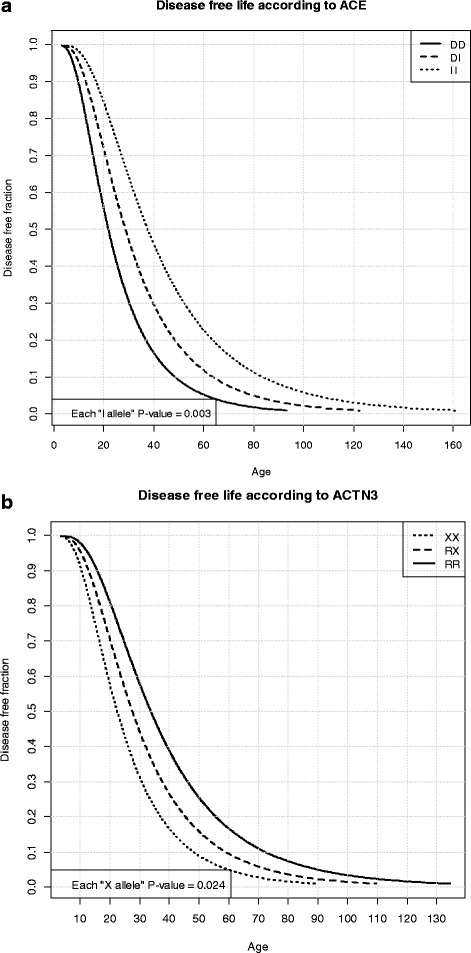
**Disease-free life according to ACE and ACTN3 polymorphisms. a)** The number of D alleles of *ACE* is significantly associated to a shorter disease-free life. **b)** The number of X alleles of *ACTN3* is significantly associated to a shorter disease-free life.

A shorter disease-free life was found, depending on the number of D alleles in the *ACE* genotype (p = 0.003, n = 85), as well as the number of X alleles in the *ACTN3* genotype (p = 0.024, n = 85) (Figure 3, a, b), while no significant association was found with *AGT* and *PPARα* polymorphisms (data not shown).

The interaction between *ACE* and *ACTN3* genotypes was non-significant in the accelerated lognormal model (p = 0.85); however, the numbers of D and X alleles were significantly associated to an earlier age of onset if included in the same survival model (p = 0.010 for the D allele and p = 0.032 for the X allele).

Table 4 shows the p values obtained when analyzing the median of the different clinical data (Walton score, 6MWT, CK and VC) in relation to the different polymorphisms.

**Table 4:** **p values observed when comparing clinical and laboratory data and the polymorphisms studied**

**Polymorphism**	**Walton** ^**§**^	Pain*	**6MWT** ^**§**^	**CK** ^**§**^	**VC%** ^**§**^
***ACE*** **I/D**	0,95	**0,028**	0,33	0,218	0,35
***ACTN3*** **R577X**	0,62	0,42	0,37	0,88	0,46
***PPAR*** **α intron 7**	0,19	0,55	0,33	0,50	0,14
***AGT*** **Met235Thr**	0,63	0,07	0,98	0,63	0,31

^§^kruskall wallis test *fisher test.

Concerning the presence of pain, the DD genotype of *ACE* was the most represented genotype among patients reporting pain (DD = 69,2%; ID = 23,8%; II = 40,0%), and there is significant difference from the genotype distribution in the whole group of patients (p = 0,028; n = 68). There is no significant association between any ACTN3 genotype and pain (p = 0,42). It is noteworthy that - among the 68 patients with available data about presence/absence of pain - the four cases with the DD/XX genotype consistently reported pain, while the four patients with the II/RR genotype did not (p = 0,029).

We did not attempt to analyze the combined *ACE-ACTN3* genotypes in relation to other clinical parameters, since the number of cases for each single genotype was too small; similarly, we did not test any other association of polymorphisms from the different genes, as the association of single genes with clinical parameters was non-significant (see Table 4).

## Discussion

Pompe disease is a monogenic autosomal recessive disorder caused by mutations in the acid alpha glucosidase gene (*GAA*, OMIM 606800)*,* which is the only gene associated to the phenotype.

Genotype-phenotype correlations have been widely discussed, and a recent paper by Kroos et al. [4] underlines the main information available. They report that genotype-phenotype correlation is strict, meaning that biallelic mutations able to completely abolish protein function cause the classic infantile Pompe disease, while the presence of at least one mutation allowing the production of a protein with some residual activity causes the late-onset forms. However, the well documented clinical variability in groups of patients with similar genotypes [14] still makes genotype-phenotype correlation an unresolved issue; modifying genetic factors are expected to influence the final clinical presentation of the disease [4].

Enzyme Replacement Therapy (ERT) is available since 2006 and data as to the variable treatment outcome among different patients have been reported [29,30], but not related to the *GAA* genotypes.

A role for modifiers genes has been reported for several diseases as Cystic Fibrosis [31], including a glycogen storage disorder, i.e. McArdle disease [32,33], and De Filippi et al. reported some data on the effect of the *ACE* I/D polymorphism on phenotype in 38 Pompe adult patients [34].

### Mutations

As recently reported by Remiche et al. [35] patients heterozygous for the c.-32-13T > G and carrying different types of severe mutations on the second allele showed a more severe phenotype.

In our study when comparing groups of patients with the same *GAA* genotypes (see results and Table 1 for details) for disease-free life (Figure 1) we observed a highly significant difference (p = 0.000163) for the c.-32-13T > G/c.1927G > A genotype, which showed an earlier age of onset, at any age, compared to other genotypes. This mutation occurs in both infantile and adult patients [36]; is reported to be associated with 1-2% of residual enzymatic activity, and with impairment in protein maturation [37]; as a barely detectable amount of the 76 kDa active protein is produced, is classified as ‘potentially less severe’ (http://www.pompecenter.nl). A structural analysis of c.1927G > A (p.G643R) mutation demonstrates that it could affect the catalytic site of the protein (see Figure 2). Though homology modelling would suggest a conservation of the protein general shape, SCpred [38] predicts G643 to be a stabilization centre, which suggests that impaired maturation could be a possible cause of reduced expression in agreement with biochemical data.

Some data about modelling for mutations other than p.G643R were reported by Sugawara et al. in [39].

Other mutations show impaired maturation with severe reduction of catalytic activity, so that overall these data alone do not explain why the c.-32-13T > G/c.1927G > A genotype is related to a significantly shorter disease-free life. Remiche et al. [35] also observed a more severe clinical presentation in their patients with the same genotype “despite the less severe qualification of this mutation”. A possible explanation is given by the study of ACE polymorphism (see below).

The c.-32-13T > G/c.525delT genotype shows in our group of patients a particular behaviour by concentrating onsets in the young-adult age interval (15 – 45 years), contrary to the other groups in which the onset of clinical symptoms may be at older ages as well. This mutation does not allow the production of any protein [40] and this biochemical information fits with the absence of very late disease onset, even in association with the mild c.-32-13T > G on the second allele. Again many other CRIM negative mutations are known, and the biochemical data alone do not explain the different behaviour of this mutation.

To better understand why a specific missense mutation as c.1927G > A, allowing the production of a small amount of protein or a non sense mutation as c.525delT CRIM negative both result in a earlier presentation of the disease, further studies as GAA activity in single cells, or analysis of binding of splicing factors or the study of production of the different isoform in muscle biopsies or in cultured muscle cells can be envisaged.

The lack of differences between VS and PLS is not surprising, as the residual activities of the two groups, extensively overlaps [3].

Other clinical parameters tested in relation to the different mutations failed to demonstrate significant differences. We are aware that these features represent, actually, “dynamic” parameters, whose values may change during the course of the disease.

Available information show that there are differences in *GAA* expression in different tissues [41], and that the pathogenesis of the disease is far more complex than previously thought [42]; moreover, *GAA* is a target for the Notch-1 signalling pathway [43] and interacts with NUMBL (http://thebiogrid.org/108823/summary/homo-sapiens/gaa.html); both Notch-1 and NUMBL genes are involved in the regulation of myogenesis [44,45].

The structural details of these interactions are not known, so the hypothesis that different mutations, even if primarily always causing glycogen storage, may have relevance for other cell functions and in turn for the final clinical phenotype, can be investigated in the future.

### Polymorphisms

Based on the differences in histopathological changes in distinct GAA knockout mice, Ponce et al. already in 1999 suggested that differences in their genetic background may play a role as a phenotype modifier and the existence of modifier genes was discussed by Kroos et al. [4].

Following previous work [32–34], we selected the polymorphisms under study (*ACE*, *ACTN3, PPARα*, and *AGT*) because of the known relevance of their genes in muscle structure to verify if any of them could act as modifier gene.

As expected (Table 3) the distribution of their genotypes did not differ from controls.

### ACE

The association between the DD genotype of *ACE* and an earlier age of onset (Figure 3a) is statistically significant and confirms our previous observation [34].

*ACE* was chosen as a candidate modifier gene since the well-known I/D polymorphism has been demonstrated to influence several muscle features such as fiber type composition [46], muscle properties [47] and sport performances [48]. ACE polymorphism also modulates the clinical presentation of McArdle disease (the D/-, DD genotypes being associated to a more severe phenotype) [34,49]; based on these observations, a clinical trial with ACE-inhibitors was started [50].

We defined the age of onset as the age at which patients experienced the first difficulties in climbing stairs/running; we believe that this information can provide data as to disease-free life and can be homogeneously collected also by different centres. The association of a shorter disease-free life with the DD genotype fits with available data on *ACE* and I/D allele function: i) the DD genotype is associated with a higher amount of circulating protein, which will cause increased levels of angiotensin II and result in vasoconstriction; our hypothesis is that, by this mechanism, a decrease in nutrients availability to working muscle would occur. ii) *ACE* may influence muscle metabolic efficiency, since an excess of II genotype was observed in elite mountaineers, and likely related to increase in type I/endurance muscle fibres [51]. The unfavourable prognostic profile of patients with the DD genotype and thus with a relative predominance of type 2 muscle fibres is in full agreement with the observation by van den Berg el at [52], that “among non-classic patients…those with early onset of symptoms tend to have more type 2 muscle fibers”.

Overall these data fit with the idea that the DD genotype may result in muscles with reduced metabolic function, and that the addition of this genotype to the background *GAA* mutations may favour an earlier clinical evidence of muscle impairment. In fact it appears relevant that three out of five of patients carrying the c.1927G > A mutation for whom a shorter disease-free life was demonstrated, (see Additional file 1: Table S1) shared the DD *ACE* genotype, and their mean age of onset was 17,6 years, while the age of onset of the two cases with ID and II were respectively 34 and 30.

The DD genotype was also associated with muscle pain (p = 0,028); pain in Pompe disease was previously reported [53] but, never addressed in detail as regards its prevalence, features, pathogenesis, and treatment options. We used anamnestic records about presence or absence of back/limbs spontaneous muscle pain (not induced by cramps or exercise-induced fatigue), collected by neurologists with high experience in caring patients with Pompe disease. Some caution must be adopted in considering this data, due to the subjective nature of this feature, and since we did not use a detailed scale for the evaluation of pain.

However, some report support a relation between pain and presence of DD alleles: In fact the relevance of the DD genotype in a microvascular disorder as diabetic nephropathy was reported in mice by Huang et al. [54] and was confirmed in a meta analysis of human data by Ng et al. [55]. Microvessel damage and reduced clearance of toxic metabolites (lactic acid?) from muscle damage (caused by vasoconstriction related to the DD genotype) is a working hypothesis for this feature.

A detailed study of the association of *ACE* and *ACTN3* polymorphisms with histochemical data obtained from muscle biopsies, are included in a manuscript in preparation.

### ACTN3

Similar results were obtained also for the polymorphism R577X in *ACTN3* gene, and the XX genotype is significantly (p = 0,024) associated with an early onset of the disease in our sample (Figure 3b).

The X allele is observed in 18% of the population and it is associated to the lack of the encoded protein α-actinin 3, which expression is restricted to fast muscle (type 2) fibers. The high frequency of *ACTN3* 577X allele in healthy controls demonstrates that the stop codon is a non-pathogenic polymorphism.

The 577X allele has been associated with better endurance performances [56], linked to type 1 (slow) fibres in which α-actinin is not expressed. The XX genotype has been reported to be connected with muscle damage after extremes performances [57], to muscle function decline with age [58], to a lower cross sectional area of thigh muscle in elderly women, and to a restricted transformation of the muscle fiber composition to type 2 fibers in response to long-term muscle disuse [59].

Overall these data indicates that XX genotype will result in impaired muscle function with age and reduced motility, and fits with our observation of shortening of disease free life in Pompe’s patients carrying this genotype.

### ACE + ACTN3

We then tested if the contemporary presence of the DD (*ACE*) and XX (*ACTN3*) genotypes, if found in the same patient, will again be associated with some of the clinical features examined. In fact (see results) the DD/XX genotype is significantly associated to a shorter disease free survival than any other combined genotypes. The two genes have completely different functions as *ACE* is relevant in renin angiotensin system and *ACTN3* encodes a muscle structural protein; these differences in function are in agreement with the observation that there is no statistical evidence for interaction between the two genes but an additive effect is present. Similarly, in a small group of patients, preliminary evidence for an additive effect of the two genotypes was observed also for pain, which was recorded in four out of four cases showing the DD/XX genotype, while pain was not reported for five cases with the II/RR genotype.

Based on these data, pain in Pompe’s patients could be tentatively interpreted as the result of muscle “damage” influenced by genetic factors (among them the polymorphisms studied) more than to the *GAA* genotype.

Other polymorphisms tested failed to demonstrate significant association with Walton score, 6MWT, CV and CK (see Table 4). The *ACTN3* XX genotype was reported to be associated with a lower CK baseline levels in healthy controls but as its contribution was only 3,5% of variation [27], so it is not surprising that we did not find a correlation for the very high CK levels in our group of patients as increased in CK levels is mainly due to muscle damage.

The observation that ACE and ACTN3 may modify age of disease onset, but not severity, in our opinion can be explained by the lack of good quantitative assessment of different clinical details of such a complex trait.

## Conclusions

Our data demonstrate that some clinical differences among patients (as age of onset or presence of pain) can be related to their genotypes when homogeneous groups of cases are compared; we expect that the study of well defined clinical features in large group of cases alike in their genetic background, will enable us to establish further genotype-phenotype correlations.

The results obtained by studying the polymorphisms in several genes whose function is associated to muscle’s behaviour show that only two of them, *ACE* and *ACTN3*, was proven to be able to modify the clinical phenotype of Pompe patients as regard to disease free life and pain. The identification of two genes able to influence Pompe phenotype clearly suggests that a large number of other genetic polymorphisms, relevant for specific clinical details showing wide variation within family and among patients, may be identified.

Our results demonstrate as the analysis of genotypic variants other than those present in the disease causing gene in patients with a well defined monogenic disorder, are worthy to be tested to obtain new prognostic data, and in the future larger series of polymorphisms can be forecasted to be used in clinical practice for a better care of patients also in relation to newly developed therapies.

## Additional file

Additional file 1: Table S1. Details of the genotypes for *GAA* mutations and *ACE, ACTN3, PPARα* and *AGT* polymorphisms far all subjects analyzed.
