# Supplementary material for: Genotype-phenotype correlation in Pompe disease, a step forward
Source: Orphanet J Rare Dis. 2014 Aug 8;9:102. doi: 10.1186/s13023-014-0102-z (PMC4249737; doi:10.1186/s13023-014-0102-z)
Supplement: Additional file 1: Table S1. — Details of the genotypes for GAA mutations and ACE, ACTN3, PPARα and AGT polymorphisms far all subjects analyzed. [file 13023_2014_102_MOESM1_ESM.doc]

| **Patient ID** | **ACE** | **ACTN3** | **PPARα** | **AGT** | **Allele 1** | **Allele 2** | **Effect** |
| --- | --- | --- | --- | --- | --- | --- | --- |
| **1** | ID | RX | GG | TT | c.-32-13T>G | 2237GA | Very severe |
| **2** | ID | RX | CC | CC | c.-32-13T>G | c.670C>T | Potentially less severe |
| **3** | II | RR | CG | TC | c.-32-13T>G | 525delT | Very severe |
| **4** | DD | RR | CG | TC | c.-32-13T>G | 525delT | Very severe |
| **5** | II | RX | CC | TC | c.-32-13T>G | c.1561G>A | Potentially less severe |
| **6** | ID | RX | CG | CC | c.-32-13T>G | [c.1833_1839del;c.1846G>T;c.1847_1848insT] | Very severe |
| **7** | ID | RR | GG | TT | c.-32-13T>G | 525delT | Very severe |
| **8** | ID | RX | GG | TC | c.-32-13T>G | c.307T>G | Potentially less severe |
| **9** | ID | RX | GG | TC | c.-32-13T>G | 2237GA | Very severe |
| **10** | DD | RX | GG | CC | c.-32-13T>G | c.1561G>A | Potentially less severe |
| **11** | ID | RX | CG |  | c.-32-13T>G | 2237GA | Very severe |
| **12** | ID | RX | CG | CC | c.-32-13T>G | 525delT | Very severe |
| **13** | DD | RR | GG | TT | c.-32-13T>G | 525delT | Very severe |
| **14** | DD | RR | GG | TC | c.-32-13T>G | delexon18 | Very severe |
| **15** | ID | RR | GG | TC | c.-32-13T>G | 2237GA | Very severe |
| **16** | ID | RR | CG |  | c.-32-13T>G | 2237GA | Very severe |
| **17** | ID | RR | GG | TC | c.-32-13T>G | 525delT | Very severe |
| **18** | II | RR | CG | TC | c.-32-13T>G | 525delT | Very severe |
| **19** | DD | RX | CG | CC | c.-32-13T>G | 1927GA | Potentially less severe |
| **20** | ID | RX | CG | TT | c.-32-13T>G | c.1124G>T | Potentially less severe |
| **21** | ID | RR | CG | TT | c.-32-13T>G | c.2530_2541del | Potentially less severe |
| **22** | ID | RR | GG | TC | c.-32-13T>G | c.1802C>G | Potentially less severe |
| **23** | ID | RX |  | TC | c.-32-13T>G | delexon18 | Very severe |
| **24** | ID | RX | GG | CC | c.-32-13T>G | c.877G>A | Potentially less severe |
| **25** | DD | RR | GG | TC | c.-32-13T>G | c.2104C>T | Potentially less severe |
| **26** | ID | RR | CG | TT | c.-32-13T>G | c.1465G>A | Potentially less severe |
| **27** | ID | RR | GG | TT | c.-32-13T>G | delexon18 | Very severe |
| **28** | ID | RX | GG |  | c.-32-13T>G | c.2219_2220delTG | Very severe |
| **29** | DD | RX | GG | TT | c.-32-13T>G | 1927GA | Potentially less severe |
| **30** | DD | RX | CC | TC | c.-32-13T>G | c.1836C>G | Potentially less severe |
| **31** | II | RR |  | TT | c.-32-13T>G | 2237GA | Very severe |
| **32** | II | RR | CG | TC | c.-32-13T>G | 2237GA | Very severe |
| **33** | II | RR |  | TC | c.-32-13T>G | 1927GA | Potentially less severe |
| **34** | DD | RR | GG |  | c.-32-13T>G | c.1465G>A | Potentially less severe |
| **35** | ID | RR | GG | TT | c.-32-13T>G | c.2219_2220delTG | Very severe |
| **36** | ID | RX | GG | CC | c.-32-13T>G | c.1655T>C | Potentially less severe |
| **37** | DD | RX | GG |  | c.-32-13T>G | c.1655T>C | Potentially less severe |
| **38** | DD | RX | CG | TT | c.-32-13T>G | delexon18 | Very severe |
| **39** | II | RR | GG | CC | c.-32-13T>G | delexon18 | Very severe |
| **40** | II | RX | CG | TT | c.-32-13T>G | c.1076-1G>C | Very severe |
| **41** | II | RX | CG | TC | c.-32-13T>G | c.1076-1G>C | Very severe |
| **42** | DD | RX | GG | TT | c.-32-13T>G | 525delT | Very severe |
| **43** | ID | RR | CC | TC | c.-32-13T>G | c.1551+1G>C | Very severe |
| **44** | ID | RR | CG | TC | c.-32-13T>G | 2237GA | Very severe |
| **45** | DD | RX | GG | TT | c.-32-13T>G | c.118C>T | Very severe |
| **46** | DD | RR | CG | TT | c.-32-13T>G | 2237GA | Very severe |
| **47** | ID | RX | CG |  | c.-32-13T>G | c.2104C>T | Potentially less severe |
| **48** | ID | RR | CG | TC | c.-32-13T>G | c.1802C>G | Potentially less severe |
| **49** | ID | RR | GG | CC | c.-32-13T>G | c.784G>A | Potentially less severe |
| **50** | ID | RX | GG | TC | c.-32-13T>G | 1927GA | Potentially less severe |
| **51** | DD | RX | CG | TC | c.-32-13T>G | 525delT | Very severe |
| **52** | ID | RR | GG | TC | c.-32-13T>G | c.1465G>A | Potentially less severe |
| **53** | ID | RX | CG | CC | c.-32-13T>G | c.1655T>C | Potentially less severe |
| **54** | II | RX | GG | TT | c.-32-13T>G | 525delT | Very severe |
| **55** | DD | RR | CG | TC | c.-32-13T>G | c.1551+1G>C | Very severe |
| **56** |  |  |  |  | c.-32-13T>G | c.1551+1G>C | Very severe |
| **57** | ID | RX |  |  | c.-32-13T>G | c.2219_2220delTG | Very severe |
| **58** | ID | RX | GG | TC | c.-32-13T>G | delexon18 | Very severe |
| **59** | II | RR | GG | CC | c.-32-13T>G | c.1802C>G | Potentially less severe |
| **60** | ID | RR | GG | TC | c.-32-13T>G | 525delT | Very severe |
| **61** | ID | RX | GG | CC | c.-32-13T>G | 525delT | Very severe |
| **62** | DD | RR | GG |  | c.-32-13T>G | 2237GA | Very severe |
| **63** | ID | RR | GG | TC | c.-32-13T>G | c.1465G>A | Potentially less severe |
| **64** | DD | RR | GG | CC | c.-32-13T>G | 2237GA | Very severe |
| **65** | II | RX | GG | TC | c.-32-13T>G | 525delT | Very severe |
| **66** | ID | RR | GG |  | c.-32-13T>G | c.1776delG | Very severe |
| **67** | ID | RX | GG | TC | c.-32-13T>G | c.307T>G | Potentially less severe |
| **68** | ID | RR | GG |  | c.-32-13T>G | 2237GA | Very severe |
| **69** | DD | RR | CG | TT | c.-32-13T>G | c.784G>A | Potentially less severe |
| **70** | DD | RR | CG | TT | c.-32-13T>G | c.784G>A | Potentially less severe |
| **71** | II | RX | CG | CC | c.-32-13T>G | 2237GA | Very severe |
| **72** | II | RX | CG | CC | c.-32-13T>G | c.1124G>T | Potentially less severe |
| **73** | ID | RX | CG | TT | c.-32-13T>G | delexon18 | Very severe |
| **74** | ID | RX | GG | TT | c.-32-13T>G | 525delT | Very severe |
| **75** | ID | RX | GG | TT | c.-32-13T>G | 525delT | Very severe |
| **76** | DD | RR | CG | TC | c.-32-13T>G | 525delT | Very severe |
| **77** | ID | RR | CG | TC | c.-32-13T>G | 525delT | Very severe |
| **78** | ID | RX | GG | TT | c.-32-13T>G | 525delT | Very severe |
| **79** | II | RX | CG | CC | c.-32-13T>G | 525delT | Very severe |
| **80** | ID | RX | GG | TC | c.-32-13T>G | c.2646del | Very severe |
| **81** | ID | RR | GG | TC | c.-32-13T>G | c.1124G>T | Potentially less severe |
| **82** | ID | RX | CG | CC | c.-32-13T>G | 525delT | Very severe |
| **83** | DD | RX | CG | TC | c.-32-13T>G | 525delT | Very severe |
| **84** | DD | RR | CC | TT | c.-32-13T>G | 1927GA | Potentially less severe |
| **85** | ID | RX | GG | TT | c.-32-13T>G | c.546+1G>T | Very severe |

Table 1 Supplementary Material.

Details of the genotypes for *GAA* mutations and *ACE, ACTN3, PPARα*  and *AGT* polymorphisms far all subjects analyzed
